# Iterative unsupervised domain adaptation for generalized cell detection from brightfield z-stacks

**DOI:** 10.1186/s12859-019-2605-z

**Published:** 2019-02-15

**Authors:** Kaisa Liimatainen, Lauri Kananen, Leena Latonen, Pekka Ruusuvuori

**Affiliations:** 1 Faculty of Medicine and Health Technology, Tampere University, Tampere, Finland; 2 Institute of Biomedicine, University of Eastern Finland, Kuopio, Finland

**Keywords:** Cell detection, Brightfield, Deep learning, Semi-supervised learning, Unsupervised domain adaptation

## Abstract

**Background:**

Cell counting from cell cultures is required in multiple biological and biomedical research applications. Especially, accurate brightfield-based cell counting methods are needed for cell growth analysis. With deep learning, cells can be detected with high accuracy, but manually annotated training data is required. We propose a method for cell detection that requires annotated training data for one cell line only, and generalizes to other, unseen cell lines.

**Results:**

Training a deep learning model with one cell line only can provide accurate detections for similar unseen cell lines (domains). However, if the new domain is very dissimilar from training domain, high precision but lower recall is achieved. Generalization capabilities of the model can be improved with training data transformations, but only to a certain degree. To further improve the detection accuracy of unseen domains, we propose iterative unsupervised domain adaptation method. Predictions of unseen cell lines with high precision enable automatic generation of training data, which is used to train the model together with parts of the previously used annotated training data. We used U-Net-based model, and three consecutive focal planes from brightfield image z-stacks. We trained the model initially with PC-3 cell line, and used LNCaP, BT-474 and 22Rv1 cell lines as target domains for domain adaptation. Highest improvement in accuracy was achieved for 22Rv1 cells. F_1_-score after supervised training was only 0.65, but after unsupervised domain adaptation we achieved a score of 0.84. Mean accuracy for target domains was 0.87, with mean improvement of 16 percent.

**Conclusions:**

With our method for generalized cell detection, we can train a model that accurately detects different cell lines from brightfield images. A new cell line can be introduced to the model without a single manual annotation, and after iterative domain adaptation the model is ready to detect these cells with high accuracy.

**Electronic supplementary material:**

The online version of this article (10.1186/s12859-019-2605-z) contains supplementary material, which is available to authorized users.

## Background

Identifying and counting individual cells from cell cultures form the basis of numerous biological and biomedical research applications [1, 2]. Determining numbers of cells reflecting the growth, survival, and death of cell populations form the foundations of e.g. basic cancer research and early drug development. Currently, the most commonly used methods for counting cells in cultures are based on either biochemical measurements, or on fluorescent stainings or markers. These methods are often either far from optimal in accuracy, costly, or time-consuming. For example, biochemical measurements are indirect measurements in terms of cell numbers. With fluorescent-based imaging, accurate cell numbers can be obtained with well-established image analysis solutions [3]. The fluorescent methods are, however, often problematic, as they require either 1) fixation and staining of cells, being costly and also limiting the number of data obtained per assay and culture, 2) live stains that are toxic to cells, limiting the time-frame of experiments [4], or 3) are based on expression of fluorescent markers in cells, severely limiting the number of cell lines available for use. In addition, the use of fluorescence requires specified imaging equipment and facilities, not at hand in all laboratories.

To avoid the need for fluorescence-based imaging, methods for brightfield imaging are used. Imaging with brightfield microscopy is straightforward with standard facilities available in almost any laboratory, and requires no labeling, making it an efficient and affordable choice. Also the drawbacks from the use of fluorophores on living cells are avoided. However, these benefits come at the cost of inferior contrast compared to fluorescence microscopy.

Most of the current brightfield-based methods rely on feature extraction from single in-focus images, or calculating the area which the cells have covered from the imaged surface. While the former works well for sparse cultures where the cells have individual profiles clearly separated from their background, these methods often do not perform well with dense cultures or cell lines with growth patterns of low contrast. Calculating the area, on the other hand, is once again an indirect estimate for cell count, and also performs more poorly the denser the cultures get. Thus, more accurate brightfield-based methods are desired for cell identification and cell number determination. Especially, improvement in identification of individual cells in dense cell clusters, as well as of cell lines with low contrast growth patterns, are required.

Various cell detection methods for brightfield images in focus have been developed in recent years [5–8]. Unfocused brightfield images or whole brightfield z-stacks have also been applied to cell detection. In our previous study, a z-stack with 25 focal planes was used as input to count PC-3 prostate cancer cells [9]. The method was based solely on the intensity values in images combined with logistic regression classifier. Selinummi et al. used z-stack for creating contrast-enhanced two-dimensional images that provided segmentation results comparable to fluorescence based segmentation [10]. Z-stacks were found to provide useful information for especially cell boundary detection. With a pinhole aperture, one can acquire unfocused images with bright spots marking the cells, which also provides results matching fluorescence based methods [11]. Ali et al. utilized unfocused images for cell and nucleus boundary detection for robust automatic segmentation procedure [12]. Their method is based on differences between two out-of-focused images from opposite directions in z-stack. A similar method with two unfocused, opposite images was proposed by Dehlinger et al. [13]. Z-stacks can also be used to handle slight variations in focal depth, which can be very useful when using autofocus algorithms. This was shown in the research performed by Sadanandan et al., where three consecutive focal planes were used as an input to deep CNN [14].

Convolutional neural networks (CNNs) are the state-of-the-art in machine learning research. After the success of CNNs in ImageNet competition [15], they have been adopted for classification tasks in biomedical imaging [16, 17]. By discarding the fully connected layers of CNN, the network becomes fully convolutional (FCN) which outputs a heatmap instead of single class value [18]. FCNs have previously been successfully used in cell detection tasks [19]. In a recent study, FCNs have been applied to class-agnostic counting using only a single training example from new domain [20]. A more sophisticated version of basic FCN is the U-Net, especially designed for biomedical image segmentation where localization has high importance [21, 22]. Usually, neural networks are trained in a supervised manner, meaning that large amounts of annotated training data is required.

Domain adaptation can solve machine learning problems where high amount of labeled training data from source domain is present, but there is only little or no labeled data for target domain [23]. Many domain adaptation methods are based on creating a transformation between source and target domains [24, 25]. Domain adaptation can also be performed by learning feature representations shared by both the source and target domains [26, 27].

We propose a method for generalized cell detection from brightfield z-stacks using single annotated cell line (PC-3) for supervised training step. U-Net is chosen as the deep learning model due to its exceptional performance in related tasks, and also due to its fully convolutional and resolution preserving nature. We test the generalization capabilities with LNCaP, BT-474 and 22Rv1 cell lines. Each of these cell lines has their own unique appearance in brightfield images, and these cell lines can be considered as target domains which are somewhat similar to the source domain. The model is trained first with annotated PC-3 samples, which results in high precision but sometimes low recall for other cell lines. We use the predictions from the pre-trained model for generating targets for unseen domains (cell lines) in unsupervised domain adaptation step. In contrast to many transfer learning approaches, we do not use any manually annotated training data for the target domain. However, we preserve some of the original training data to prevent excessive influence of the imperfections in predictions. Thus, training is performed in semi-supervised manner while domain adaptation is unsupervised.

## Methods

In this study, we explore methods for improving generalization in cell detection. We use brightfield z-stacks of PC-3 cell line for supervised training of U-Net-based model for cell detection, and apply an unsupervised domain adaptation step to improve the detection accuracy of cell lines lacking annotated training data. Implementation was programmed with Python language and Keras and TensorFlow modules for deep learning.

### Brightfield data

The data consists of brightfield focus stacks (a.k.a. z-stacks) of monolayer cultures of cancer cell lines. Images were acquired with QImaging Retiga-2000R camera using Olympic IX71 microscope and Objective Imaging Surveyor scanning and imaging software. The z-stack range was 240 *μm*, and distance between adjacent focal planes was 10 *μm*. Images have a pixel resolution of 1596×1196 pixels, corresponding to an area of 1190.8*μm*×891.4*μm*. Autofocus method was used to detect most focused image, above and below of which 12 focal planes were imaged. Thus, each stack consists of 25 focal planes in which the 13th focal plane is in focus.

**Fig. 1:**
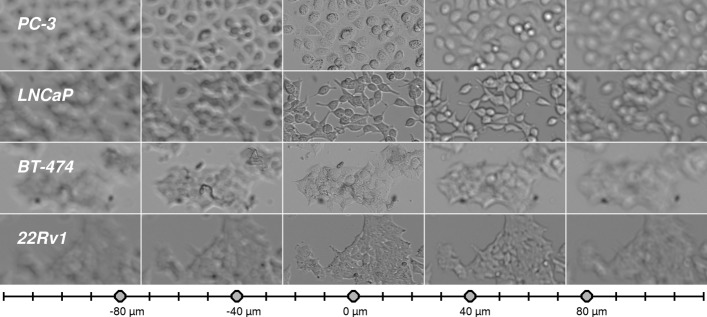
Examples of each studied cell line in z-stack with 25 focal planes. The grid represents focal planes, circles marking the planes in the figure in corresponding order

Four cancer cell lines were used in this research: prostate cancer cell lines PC-3, 22Rv1 and LNCaP, and breast cancer cell line BT-474. All cell lines were obtained from American Type Culture Collection (ATCC, Rockville, MD, U.S.A.) and cultured under the recommended conditions. Example images from these cells are shown in Fig. 1. These cell lines were chosen due to their differential appearance in z-stacks, varying from separately growing, high contrast PC-3 and LNCaP to dense and low contrast populations of BT-474 and 22Rv1. The networks were trained with PC-3 using the same data that was used in our previous study [9]. This data was acquired from one cell cultivation, where the cells grew for six days and were imaged daily. For training, we used two images from each day. Thus, twelve images of size 1196×1596, including 5878 annotated cells in total were used.

Four images from each cell line were annotated for the purpose of validating results. The annotated cell count in testing data is 1975 for PC-3 cells, 2183 for LNCaP cells, 1022 for BT-474 cells and 2883 for 22Rv1 cells. Annotations for validating PC-3 cells were not used in training, and the images of PC-3 cells for validation are from a separate cultivation than the training data. In the domain adaptation step, none of the annotated images of other cell lines were used to prevent any distortion of results from over-fitting.

### Method selection and comparison

When selecting the best method for this task, the first criterion was that the resolution of prediction has to be similar to input image resolution, to ensure best possible separation of the cells. Second criterion was that the prediction should be performed to the whole image at once, since pixelwise prediction would require excessive computational resources when fulfilling the resolution requirement. Thus, many deep learning architectures were discarded. Deep learning architectures similar to U-Net fulfill these requirements. Also other, more basic fully convolutional networks without maxpooling layers were taken into consideration, however, the results were inferior to U-Net-like architectures. Two chosen methods from literature were included for comparison: subtraction between opposite z-stack planes [12], and image processing based method from one unfocused image [7]. Some experiments were also performed with pixel intensity based logistic regression classification [9], but the results for other cell lines than PC-3 were not comparable to other methods.

In addition to the actual method, the most suitable focal planes from z-stack were defined. Each method was tested with various degrees of unfocusing and, if possible, various amount of input focal planes. The results of these experiments are shown in Table 1.

**Table 1:** Method and input comparison. For methods by Ali [12] and Buggenthin [7] the results from focal plane producing best overall score are given

	Ali	Buggenthin	U-Net	U-Net	U-Net	U-Net	U-Net (smaller)
Focal planes	8,18	9	13	11-15	12-14	13-15	13-15
PC-3	0.90	0.8	0.933	0.949	0.944	**0.955**	**0.955**
LNCaP	0.683	0.673	0.885	0.889	0.88	0.893	**0.895**
BT-474	0.576	0.671	0.76	0.724	**0.777**	0.76	0.757
22Rv1	0.511	0.606	**0.69**	0.612	0.619	0.673	0.672
Total	0.67	0.688	0.817	0.794	0.805	**0.82**	**0.82**

Best result for each cell line is marked with boldface

Best overall results were acquired with U-Net architecture taking focal planes 13, 14 and 15 as input. Example images of these input focal planes for each cell line are shown in Additional file 1. Most focused focal plane in z-stack has index 13 according to autofocus algorithm used in imaging. However, when looking at the planes in question, one can argue that actually the most focused focal plane is the one with index 14. Then, our conclusion for best suited planes would be the same as in [14].

**Fig. 2:**
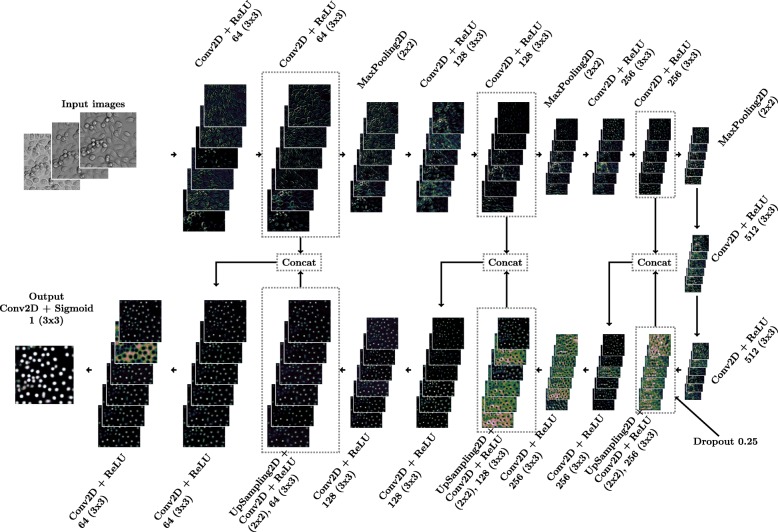
Architecture of the network. The network is a reduced version of U-Net, with one set of layers removed to maintain symmetry of the U-Net. Image patches are real examples of inputs and outputs, selected by maximum activation. More intermediate outputs are presented in Additional file 1. Each image patch is normalized for better visibility before adding to stack of patches

When studying the intermediate outputs of U-Net model, we noticed that cell detections were already present in multiple intermediate layers before the last. Thus, the question rose whether some of the layers could be discarded without loosing accuracy. One residual layer set was removed from the architecture while keeping the symmetry, and in the last column of Table 1 we can see that the results are as good as with whole U-Net. Thus, to reduce computational burden and memory requirements of the model, this smaller architecture was chosen as the method. This network architecture, with some example layer outputs, is shown in Fig. 2. More detailed description of layers is presented in Additional file 1. After selecting the best method, the training pipeline was further improved for better accuracy.

### Training details

Initially, we trained the deep learning model with PC-3 exclusively. For training target, a binary mask was created. Each cell is presented with a disk shaped structuring element with a radius of 8, but if these circles touch each other, the radius is reduced for better separation of the cells. The maximum radius of 8 is a good trade-off between cell separation capability of the model and class balance in training. With this radius, we get approximately 20 to 1 relation between background and cell pixels, which is still a manageable class balance. We use a total of 12 1596×1196 images for training the network. One quarter of the training set was set aside for validation during training.

The most difficult cell line from cell detection point-of-view is 22Rv1. These cells are much smaller than the cells from other cell lines. Based on this knowledge, we augmented the training data by resizing PC-3 images to 75 percent of their original size, which doubled the amount of training data. To match the resized images, maximum radius for circles in training targets was reduced to 6 pixels, using the same proportion.

Since training target is a binary mask, binary crossentropy was chosen as a loss function. We used stochastic gradient descent (SGD) optimizer with a Nesterov momentum of 0.8, and batch size was 5 samples. We set the learning rate to 0.1 at the beginning of training, and after every 10 epochs reduced it to half. Since convolution discards a small proportion of information from the border of the image, and the fully convolutional nature of U-Net architecture allows changing input size, the size of input patches was switched after each set of 10 epochs for better utilization of the training data. After loading each training set, image transformations were applied randomly to each patch of training data. These transformations include rotation, translation, small intensity shifts and adding noise. The model was trained for a total of 60 epochs. However, the weights were saved only when validation loss decreased, resulting in an actual amount of epochs less than 60.

### Domain adaptation

In this study, domain adaptation is used to fit the model trained with PC-3 cell line (source domain) to other cell lines (target domains). Not a single cell from target domains LNCaP, BT-474 and 22Rv1 was annotated for training purposes.

**Fig. 3:**
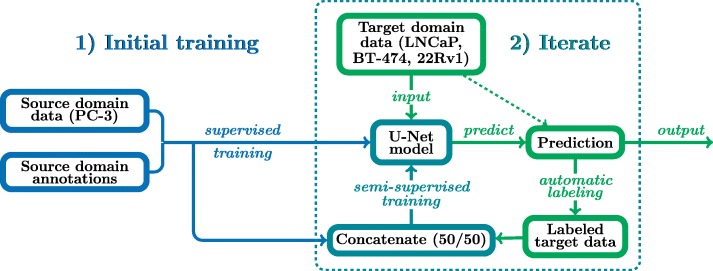
Pipeline for iterative unsupervised domain adaptation for cell detection

After achieving reasonable recall and, more importantly, high precision for all cell lines via training with only PC-3, the domain adaptation step was applied. In Fig. 3, the pipeline of the method is presented. Domain adaptation is marked with a blue dashed square and it is repeated six times in total. Domain adaptation was performed in an unsupervised manner; half of the training data was generated by auto-labeling from the target cell line, while the other half represented randomly selected patches of previously used PC-3 training data. Since the domain adaptation step is based on predictions of unseen cell lines, annotated PC-3 training data is required to prevent the model from fitting to false positives or negatives. Without the annotated data, the false predictions can get amplified during training since auto-labeling is performed iteratively during training.

Auto-labeling was performed in the following manner. First, prediction was calculated for four randomly selected images of the target cell line. The images that were annotated for testing purposes were not available for the selection. Then, local peaks were detected from the predicted heat map with a threshold of 0.2 and a minimum distance of 5 pixels between peaks. Threshold was set low to include also weak predictions to training data. Each peak was marked as a cell, and these cell points were transformed to training targets with binary dilation with a disk shaped structuring element with a radius of 6 pixels. The model was trained for another 60 epochs in total. New prediction-based training data was calculated after each set of 10 epochs, simultaneously decreasing the learning rate to half and changing patch size, as was performed in supervised training.

### Validation metrics

In the task of cell counting, true negatives are ambiguous. Since true negatives are not included in F_1_-score, it is a suitable metric for validating our results. To find matching cells between prediction and ground truth, detected cell coordinates were compared to ground truth coordinates with Euclidean distance, and distance below 20 was considered as detection. If multiple coordinates were found within the threshold distance, only the closest of these was accepted as detection.

We count true positives (*TP*), false positives (*FP*) and false negatives (*FN*) for each image in the test set. With these values, we calculate precision (positive predicting value), $ \frac {TP}{TP+FP}$, and recall (sensitivity), $ \frac {TP}{TP+FN}$. Finally, F_1_-score is calculated via equation $2 \times \frac {precision \times recall}{precision + recall}$.

In density-based accuracy calculations, a density map was created using kernel density estimation with normal kernel (*σ*=50 pix) for smoothing discrete cell locations into local neighborhood. This density map was then divided into five areas covering equal density range. Note that this does not correspond to dividing the areas based on equal area coverage, nor will there be equal number of cells within the density areas.

## Results

To demonstrate the performance of the proposed cell detection methodology, we present results for different experimental setups. First, we show how deep learning masters the challenge of label-free cell detection from bright field focus stacks in a very accurate manner. Second, we show how precision remains high for cell lines never seen by the classifier. Finally, we present how the high precision can be used for iterative unsupervised domain adaptation. Furthermore, we present detection accuracy in relation to cell growth density. While small example images are presented in result figures, examples of whole image level detections are given in Additional file 1.

**Fig. 4:**
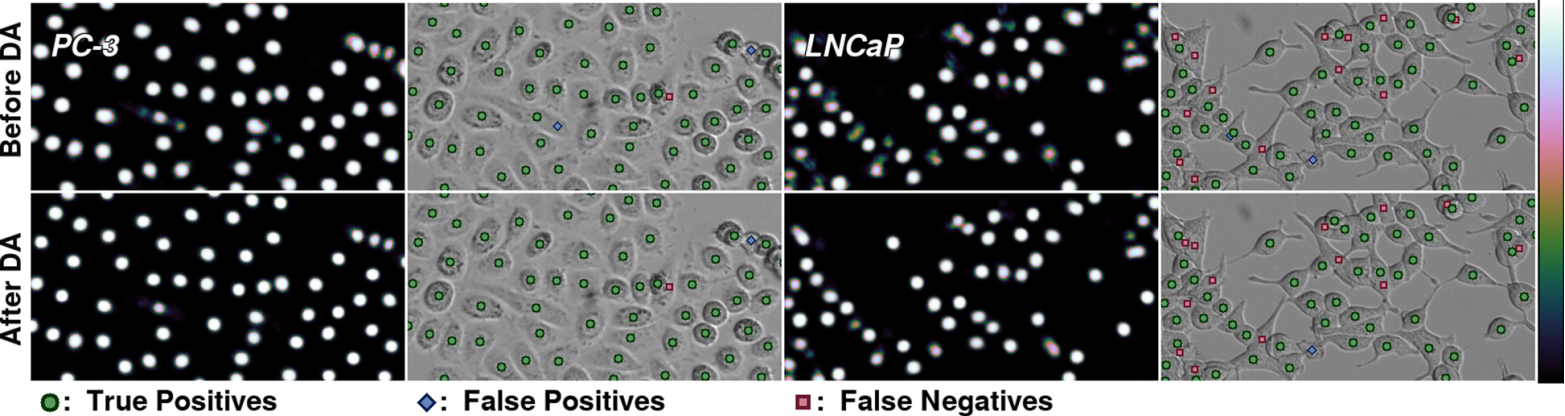
Unprocessed predictions and detected cells of PC-3 and LNCaP cell line. The figures in upper row are results before domain adaptation, and the bottom row shows the very similar results after domain adaptation step with corresponding cell line. The heat maps present unprocessed results of detection. Results are presented with cubehelix colormap [28]

We performed convolutional neural network based label-free cell detection of PC-3 cancer cells. We acquire F_1_-score of 0.95 for this cell line. An example prediction is shown in Fig. 4, on the left half. Even though the prediction is often close to perfect, the stacking cells are not always well separated. This slightly reduces the overall accuracy, but with a score of 0.95, our method can still be used to e.g. accurately count the growth curve of PC-3 cell line.

Then, we tested how the cell detector generalizes from PC-3 to multiple cancer cell lines. The scores acquired with the model trained with PC-3 cell line only are shown in first halves of plots in Fig. 6. We achieve a high accuracy with 0.89 F_1_-score for LNCaP cell line. An example detection is shown in Fig. 4. When moving to cell lines of dense populations and low contrast, namely BT-474 and 22Rv1, the scores are decreased. Precision still remains high for both of these cell lines, but recall drops drastically when compared to LNCaP and PC-3. Also, recall fluctuates a lot between each set of 10 epochs. We could say that the model does not actually fit to BT-474 and 22Rv1 as it does to PC-3 and LNCaP. Indeed, the best score is acquired after only 20 epochs of training, which implies that the more the model is fitted towards PC-3, the farther it goes from fitting to BT-474 and 22Rv1. At this stage, heavy data augmentation had already been applied and this improved especially the 22Rv1 detection. For this cell line, the F_1_-score is about 0.1 higher when we double the training data by resizing it to 75 percent of the original size. 22Rv1 cells are the smallest in our set, so smaller cells in training data naturally improve the accuracy.

**Fig. 5:**
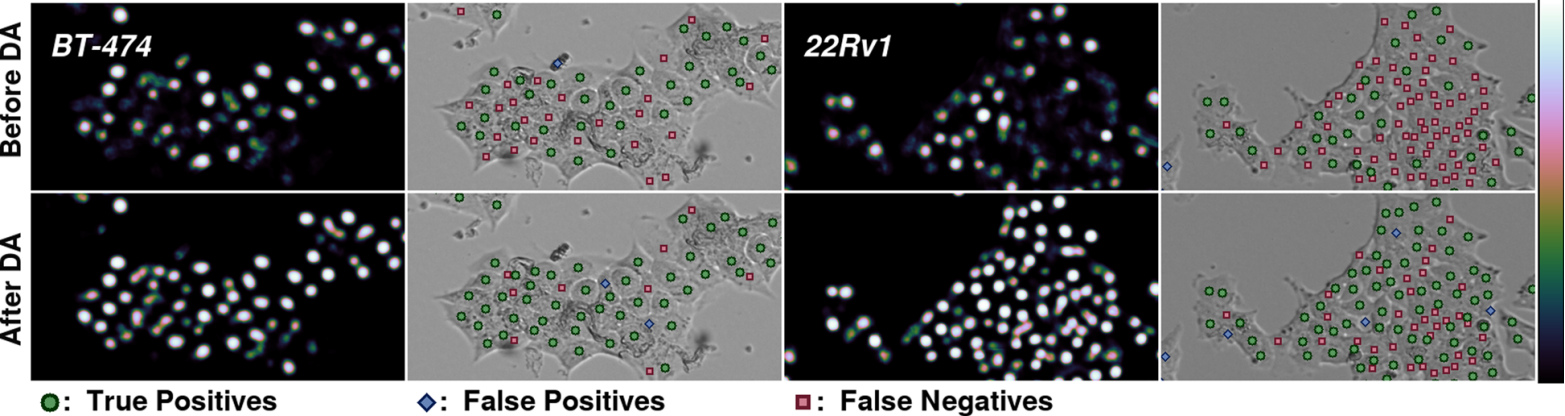
BT-474 (left) and 22Rv1 (right) detections before (top) and after (bottom) domain adaptation step

Next, we applied unsupervised domain adaptation to improve generalization to unlabeled data from unseen cell lines. In Fig. 5, we show how domain adaptation step improves the results. F_1_-score for 22Rv1 rises from 0.65 to 0.84, and the score for BT-474 rises from 0.74 to 0.87.

**Fig. 6:**
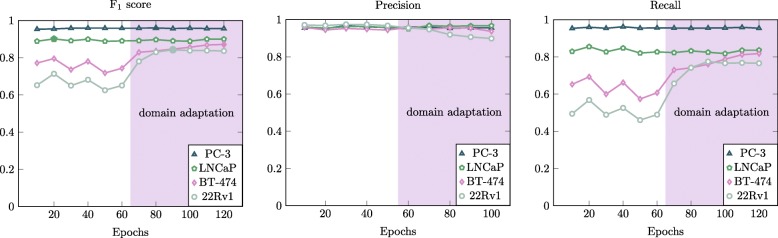
F_1_-score, precision and recall as a function of training epochs for all cell lines. First 60 epochs (x-axis) the model trained with PC-3 only, and next 60 epochs the model was trained also with the corresponding cell line

**Fig. 7:**
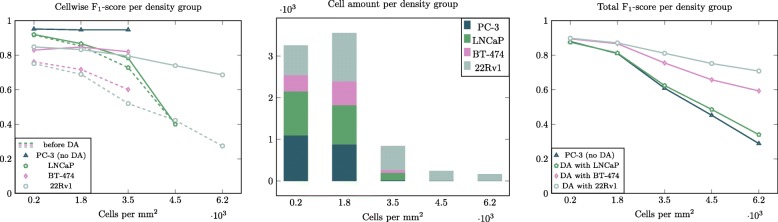
Accuracy for cell lines before (dashed) and after domain adaptation with corresponding cell line (left). No score is given when cell line is not present in density group. Amount and type of cells in density groups (center). Accuracy for whole test set, including all cell lines (same set for all models), calculated with models before domain adaptation and after adapting to each unseen domain (right)

In Fig. 7, we show detection accuracies divided into groups of different cell densities, and cell amount within those areas. Density areas are illustrated as contour overlays in Additional file 1. Accuracies are calculated for models adapted to each unseen domain, and also for the model before domain adaptation. In Fig. 7, left panel, the dashed lines represent accuracy before domain adaptation. The accuracy decreases considerably when moving to denser areas, but after domain adaptation (solid lines), the accuracies of the densest areas are comparable to sparser areas. It should be noted that in the second to last densest group, there are only 4 LNCaP cells, which results in sudden drop in accuracy.

## Discussion

### Convolutional neural network based label-free cell detection of PC-3 cancer cells

The convolutional neural network based label-free cell detection was applied to data from PC-3 cancer cell line with the accuracy of F_1_-score 0.95. PC-3 cell line has a clear profile in brightfield focus stacks and high detection accuracy is acquired with as few as 10 to 20 epochs of training of a deep learning model, as shown in Fig. 6. With a U-Net-like model, we obtain a clean and sharp heat map as an output, where each cell is represented with a circle. It should be noted that we aim at cell detection, not segmentation, and the model outputs circular detections since it was trained with a binary mask where circles represent the cells. A high accuracy can be achieved also with a single image in focus, but with z-stacks we can improve especially the precision of detections. Non-cell objects are often not as similar to cells in images out-of-focus as they are in only focused image. For example, the artifacts caused by impurities in camera lens do not change their appearance when going out of focus.

### Generalization from PC-3 to multiple cancer cell lines

Generalization to other cell lines was analyzed by applying the cell detection model trained with PC-3 cell line to data from other cancer cell lines, which were LNCaP, BT-474 and 22Rv1. In brief, the results obtained for LNCaP were of high accuracy, while the accuracy dropped for BT-474 and 22Rv1. The LNCaP cell line somewhat resembles PC-3 since the cells tend to grow separately. Especially the cell lines that grow in dense populations do not receive very high accuracy with the network trained with PC-3 only. One reason for this might be that there often is no background around cells that grow close to each other, while each non-stacked cell of PC-3 has at least some background around them. Also the height and shape of the cells in z may affect their contrast properties. In Fig. 5, on top row, we see detections of BT-474 and 22Rv1 after supervised training. BT-474 is detected with reasonable accuracy, achieving an F_1_-score of 0.74. For the dense population of 22Rv1 cells, most cells in the center of the example image have not been detected, and the F_1_-score is only 0.64. However, the score is high enough for successful domain adaptation.

### Improved generalization through iterative unsupervised domain adaptation

With domain adaptation, domain being another cell line, we can greatly increase the accuracy of the cell detection for the unseen cell lines, especially those growing in dense populations. The worse the accuracy is before domain adaptation, the more it is improved with domain adaptation. In Fig. 5, we show how multiple previously undetected cells get clear detections after domain adaptation (bottom row). Especially in 22Rv1 (Fig. 5 on the right), the improvement is drastic. Even though some of the cells in the dense center have non-zero confidence that is not registered to the score before domain adaptation, there are several clear detections for these cells after domain adaptation (compare top and bottom row).

For cell lines that already get very accurate predictions with F_1_-scores around 0.9 after initial training with PC-3, the domain adaptation step does not result in a significant change. Thus, we can apply the domain adaptation step to any cell line with reasonable confidence of not reducing the detection accuracy. In Fig. 4, we can see very little difference in the detections for PC-3 and LNCaP cells, even though in the predicted heat map, the detection signals are visibly more distinct.

### Relation between accuracy and cell growth density

In order to gain more insight into the effect of cell density on the detection accuracy, we created a pooled test set by using data from all cell lines for determining the detection accuracy. It should be noted that this time, the absolute number of cells and the relative fraction of cells from each cell line varies from density area to another (see Fig. 7, center panel for the cell type distributions among areas). From results for the whole test set (Fig. 7, right panel), we see that with the model trained with PC-3 only, the accuracy is low on dense areas. In addition, when adapting to LNCaP domain, the score remains low although slight improvement is apparent. When training with the densely growing cell lines, BT-474 and 22Rv1, the scores are considerably improved. Even though both of these cell lines grow in dense populations, the BT-474 cells are bigger than 22Rv1 cells. Thus, 22Rv1 cell line is able to grow more dense than the other cell lines, resulting in that they are the only cell line present in densest of areas (Fig. 7, center panel). Yet, the improvement in scores for BT-474 is comparable to 22Rv1-trained model. This implies that the size of cells is not a property that greatly differentiates the cells in the model’s point of view. However, the height of cells affects the contrast of cells in z-stacks. This is a property that also affects the similarity between cell lines. In addition, the cells within dense populations do not have any visible background surrounding them, which is a joint property of BT-474 and 22Rv1 cell lines.

According to these results, the overall accuracy never decreases when adapting to a new domain. Thus, the new features learned during domain adaptation cannot be just cell line specific. In addition, since PC-3 data is also used when adapting to a new domain, the PC-3 detection accuracy does not decrease.

## Conclusions

Many applications of biological and biomedical research require accurate cell detection and counting. Our results show that with deep learning we can accurately detect PC-3 cells from brightfield z-stacks without the need for fluorescence imaging. Furthermore, the model generalizes well for cell lines similar to PC-3. In case of densely growing cells with low contrast, properties that differentiate these cells from PC-3, we achieve lower recall but high precision. High precision enables automated generation of suitable training targets for domain adaptation. With iterative unsupervised domain adaptation, we can increase the accuracy of previously poorly detected cell lines considerably. The higher the dissimilarity is between the source and the target cell lines, the more improvement can be achieved via domain adaptation.

Our contribution to research fields depending on cell counting is a framework for unsupervised domain adaptation, including a pre-trained model, for accurate detection of various cell lines unseen by the classifier. Manual annotation for these cell lines is not required due to automated labeling of new training data. Since our method is based on brightfield images, it is available for all laboratories with just basic imaging equipment.

## Additional file

Additional file 1 Supplementary **Figures S1–13** and Supplementary **Table S1**. (PDF 18200 kb)

## Abbreviations

CNN: Convolutional neural networks
FCN: Fully convolutional network
FN: False negative
FP: False positive
TP: True positive
