# Supplementary material for: Iterative unsupervised domain adaptation for generalized cell detection from brightfield z-stacks
Source: BMC Bioinformatics. 2019 Feb 15;20:80. doi: 10.1186/s12859-019-2605-z (PMC6376647; doi:10.1186/s12859-019-2605-z)
Supplement: Supplementary file 1 — Supplementary Figures S1–13 and Supplementary Table S1. (PDF 18200 kb) [file 12859_2019_2605_MOESM1_ESM.pdf]

Iterative semi-supervised domain adaptation for generalized cell detection from brightfield z-stacks
Supplementary material

Kaisa Liimatainen, Lauri Kananen, Leena Latonen, Pekka Ruusuvuori

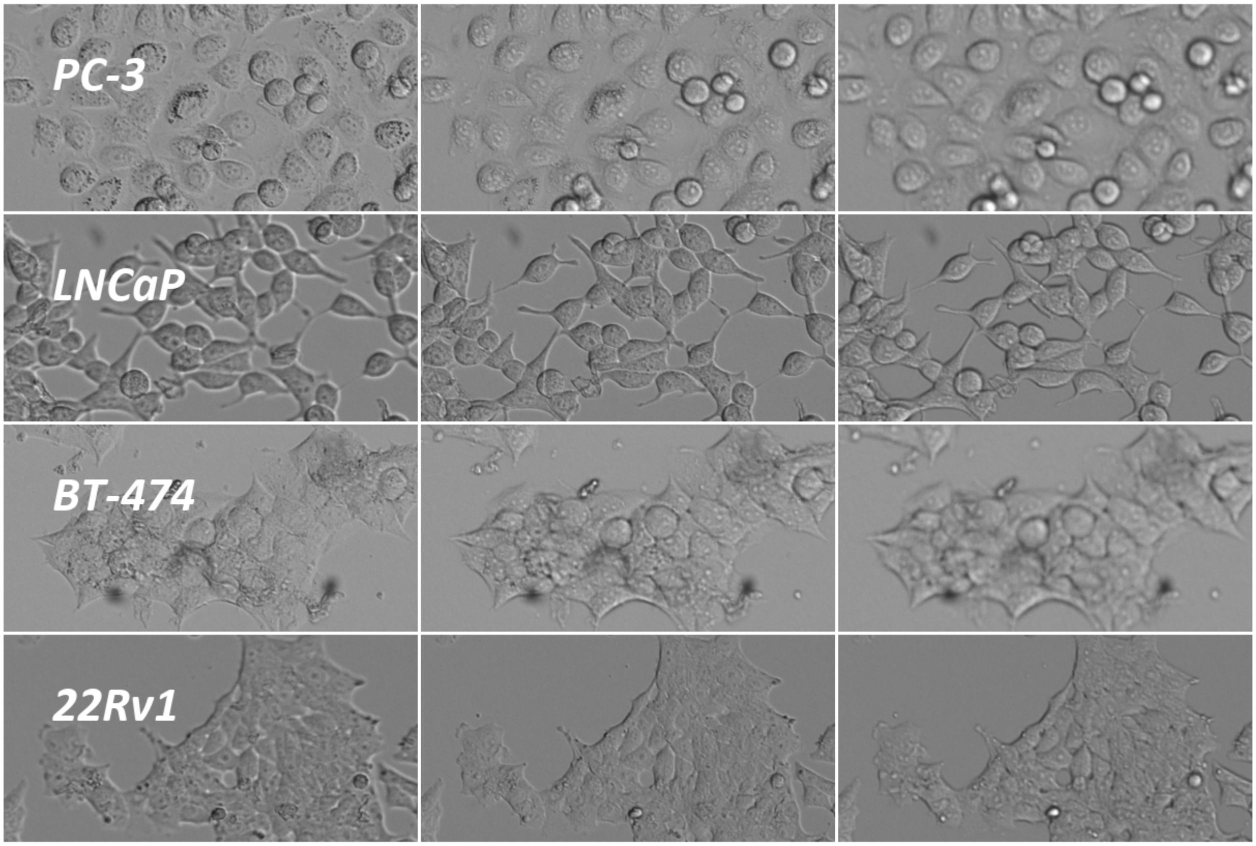

Supplementary Figure 1: Model input examples for each cell line. Input focal planes are at indices 13 (in focus, left), 14 (center) and 15 (right).

Supplementary Table 1: Detailed network description. Model is based on U-Net architecture, with one "set" of layers removed from both sides of the bottleneck layers (conv4x), thus keeping the symmetry. Layer "sets" are separated with horizontal line. Outputs of layers with colored rows are presented in following figures.

Layer type	Layer name	Input layer	Kernel size	Kernel amount	Output size	Activation
Input	input				(1,128,128,3)	
Conv2D	conv11	input	(5,5)	64	(1,128,128,64)	ReLU
Conv2D	conv12	conv11	(5,5)	64	(1,128,128,64)	ReLU
MaxPooling2D	maxp2	conv12	(2,2)		(1,64,64,64)	
Conv2D	conv21	maxp2	(3,3)	128	(1,64,64,128)	ReLU
Conv2D	conv22	conv21	(3,3)	128	(1,64,64,128)	ReLU
MaxPooling2D	maxp2	conv22	(2,2)		(1,32,32,128)	
Conv2D	conv31	maxp2	(3,3)	256	(1,32,32,256)	ReLU
Conv2D	conv32	conv31	(3,3)	256	(1,32,32,256)	ReLU
MaxPooling2D	maxp3	conv32	(2,2)		(1,16,16,256)	
Conv2D	conv41	maxp3	(3,3)	512	(1,16,16,512)	ReLU
Conv2D	conv42	conv41	(3,3)	512	(1,16,16,512)	ReLU
Dropout (0.25)	drop4	conv42			(1,16,16,512)	
UpSampling2D	upsa5	drop4	(2,2)		(1,32,32,512)	
Conv2D	conv5	upsa5	(3,3)	256	(1,32,32,256)	ReLU
Merge (concat)	merge5	(conv32,conv5)			(1,32,32,512)	
Conv2D	conv51	merge5	(3,3)	256	(1,32,32,256)	ReLU
Conv2D	conv52	conv51	(3,3)	256	(1,32,32,256)	ReLU
UpSampling2D	upsa6	conv52	(2,2)		(1,64,64,256)	
Conv2D	conv6	upsa6	(3,3)	128	(1,64,64,128)	ReLU
Merge (concat)	merge6	(conv22,conv6)			(1,64,64,256)	
Conv2D	conv61	merge6	(3,3)	128	(1,64,64,128)	ReLU
Conv2D	conv62	conv61	(3,3)	128	(1,64,64,128)	ReLU
UpSampling2D	upsa7	conv62	(2,2)		(1,64,64,128)	
Conv2D	conv7	upsa7	(3,3)	64	(1,128,128,64)	ReLU
Merge (concat)	merge7	(conv12,conv7)			(1,128,128,128)	
Conv2D	conv71	merge7	(3,3)	64	(1,128,128,64)	ReLU
Conv2D	conv72	conv71	(3,3)	64	(1,128,128,64)	ReLU
Conv2D	conv8	conv72	(3,3)	1	(1,128,128,1)	Sigmoid

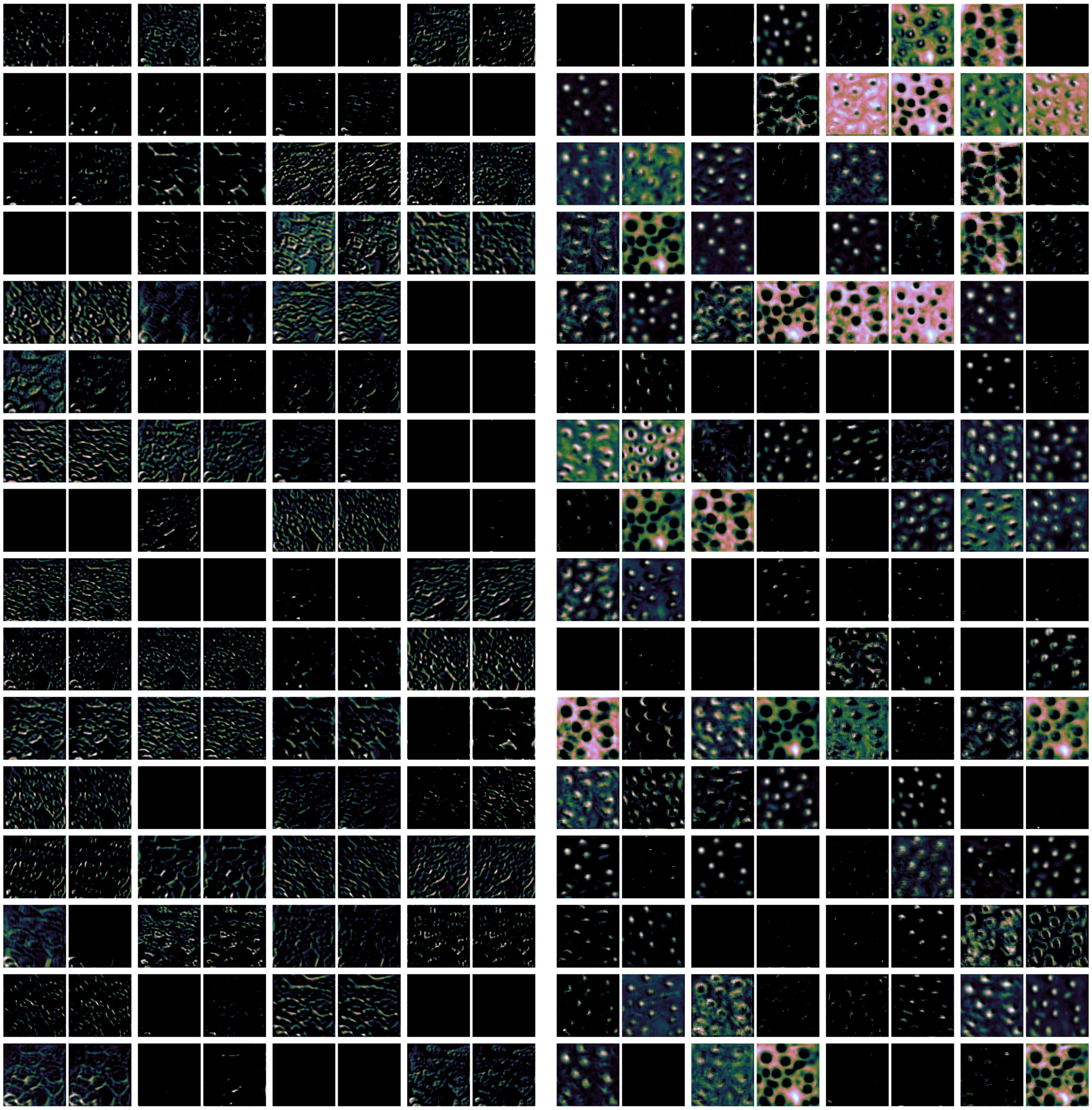

Supplementary Figure 2: Normalized output pairs of layers conv12 (left) and conv7 (right) before and after domain adaptation with 22Rv1. Input image was from 22Rv1 cell line. These layers are the inputs for last merge layer (merge7). In following output images the same models and input are used.

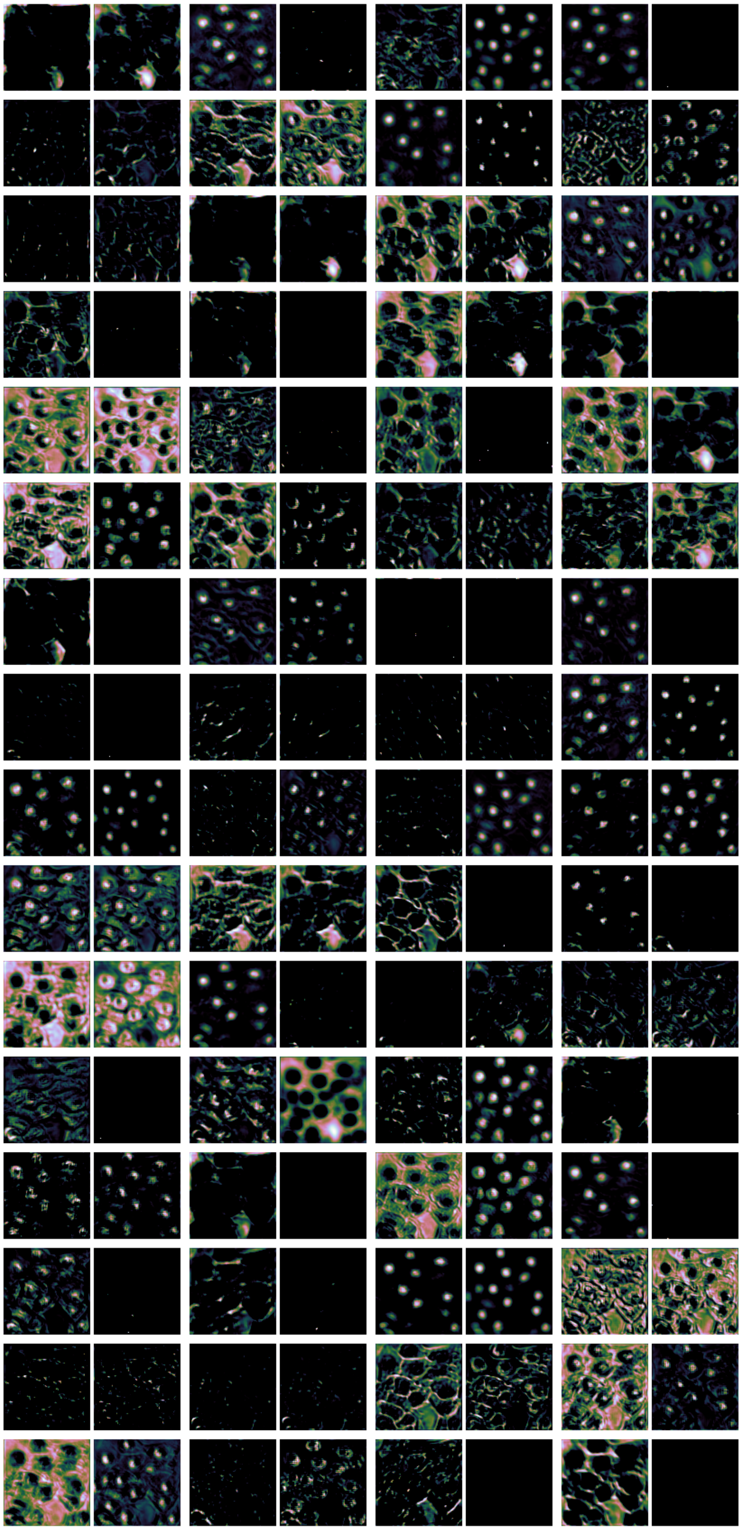

Supplementary Figure 3: Normalized output pairs for layer conv71, the convolutional layer following last merge layer (merge7). Inputs for merge7 layer were shown in Supplementary Figure 2.

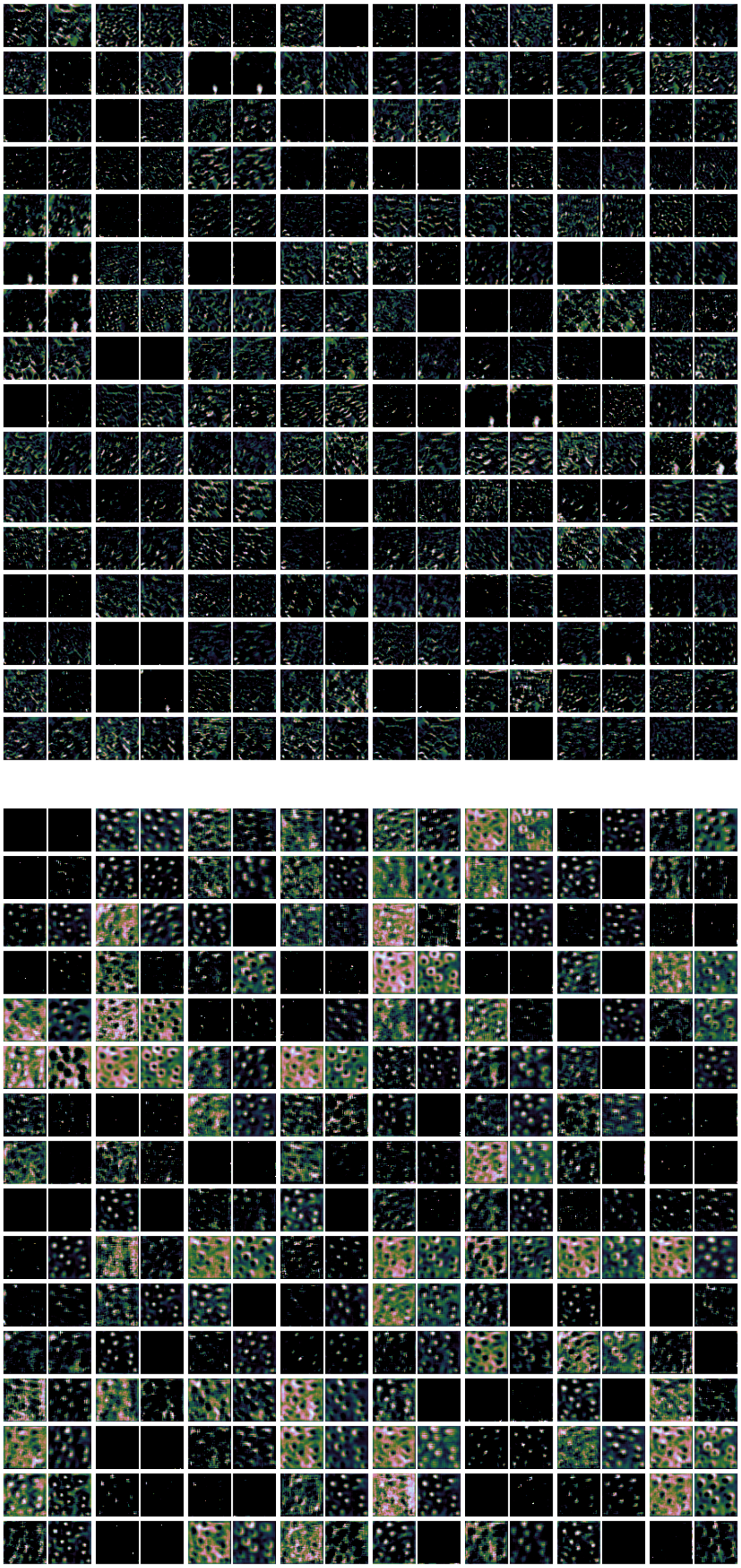

Supplementary Figure 4: Normalized output pairs of layers conv22 (top) and conv6 (bottom). These layers are the inputs for middle merge layer (merge6).

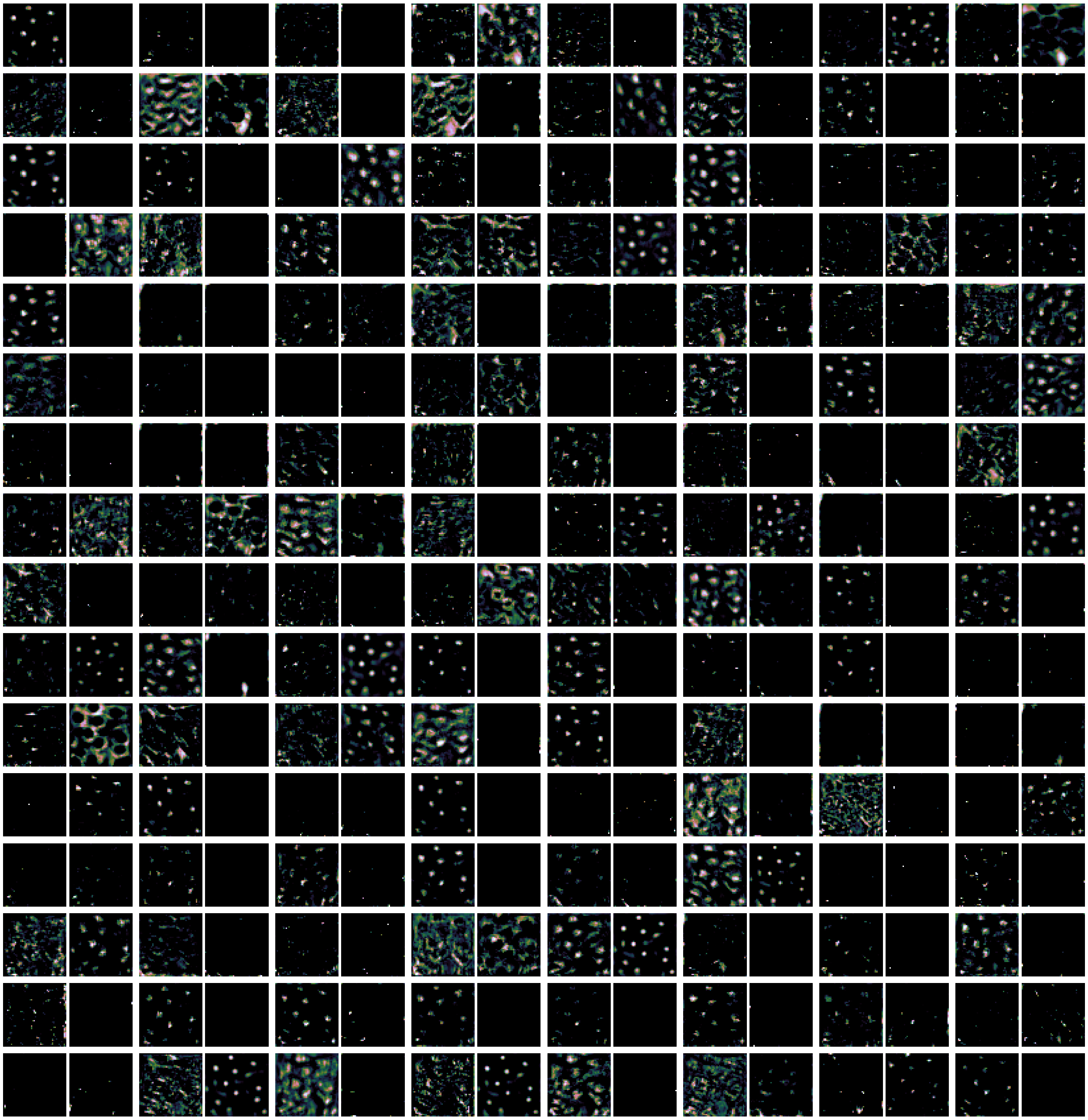

Supplementary Figure 5: Normalized output pairs for layer conv61, the convolutional layer following merge6 layer. Inputs for merge6 layer were shown in Supplementary Figure 4.

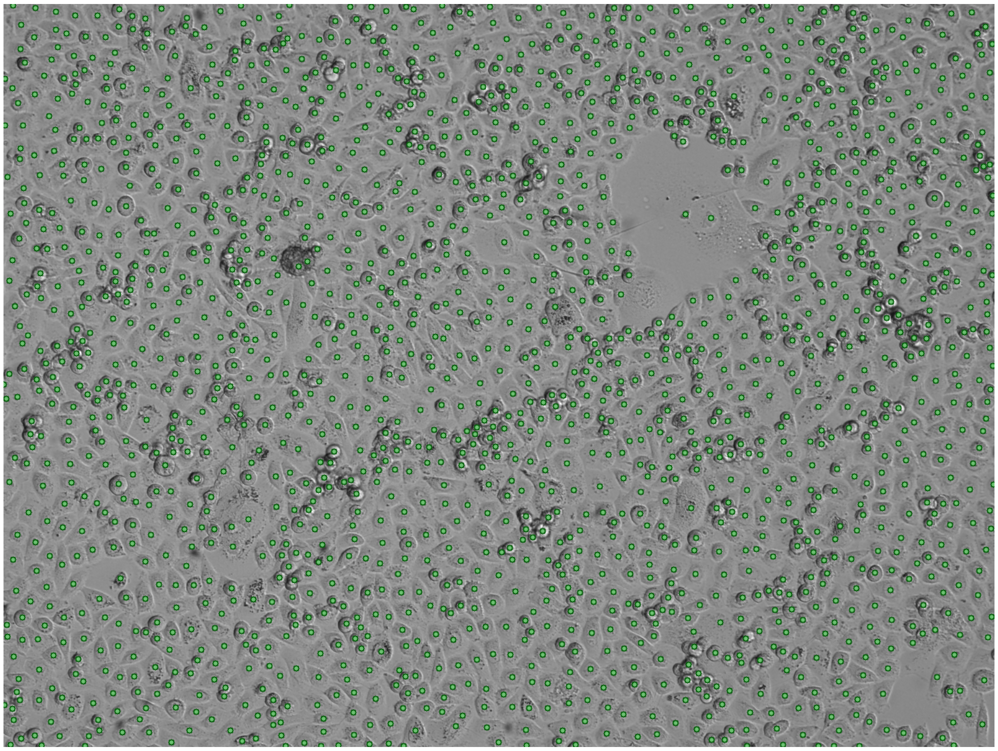

Supplementary Figure 6: PC-3 detections in crowded population.

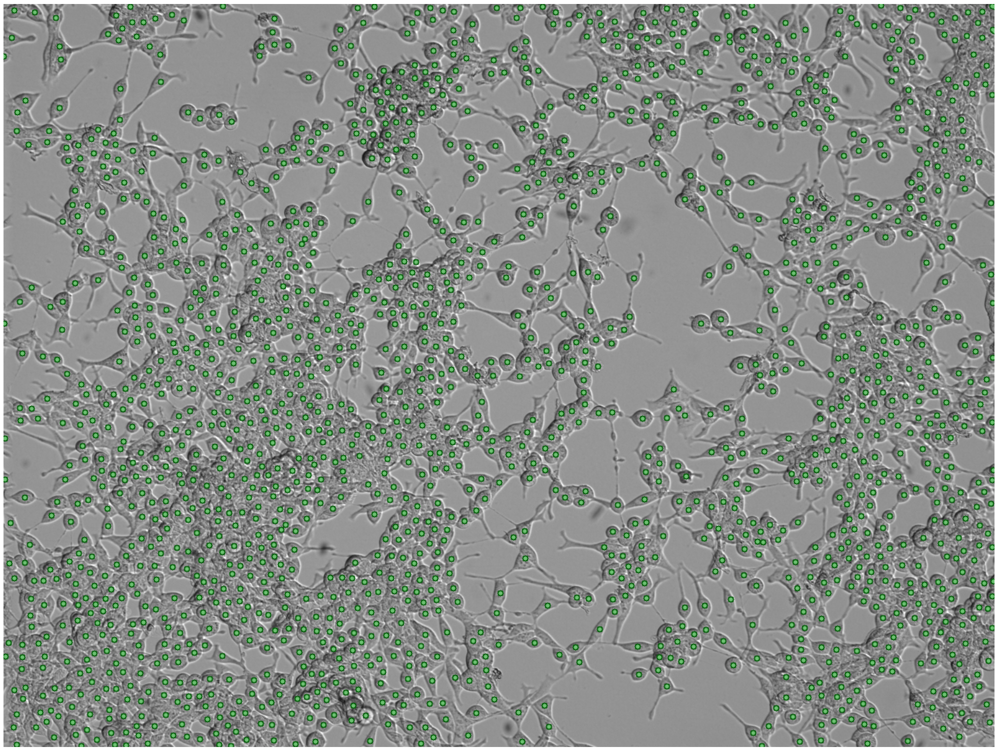

Supplementary Figure 7: LNCaP detections in crowded population (after domain adaptation).

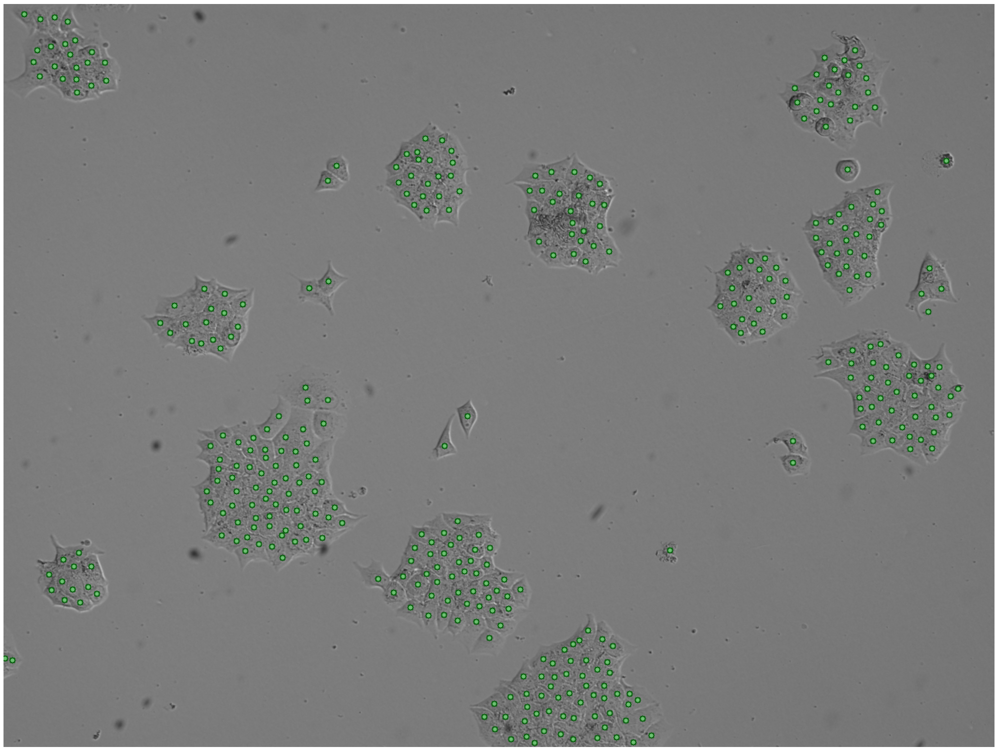

Supplementary Figure 8: BT-474 detections in crowded population (after domain adaptation).

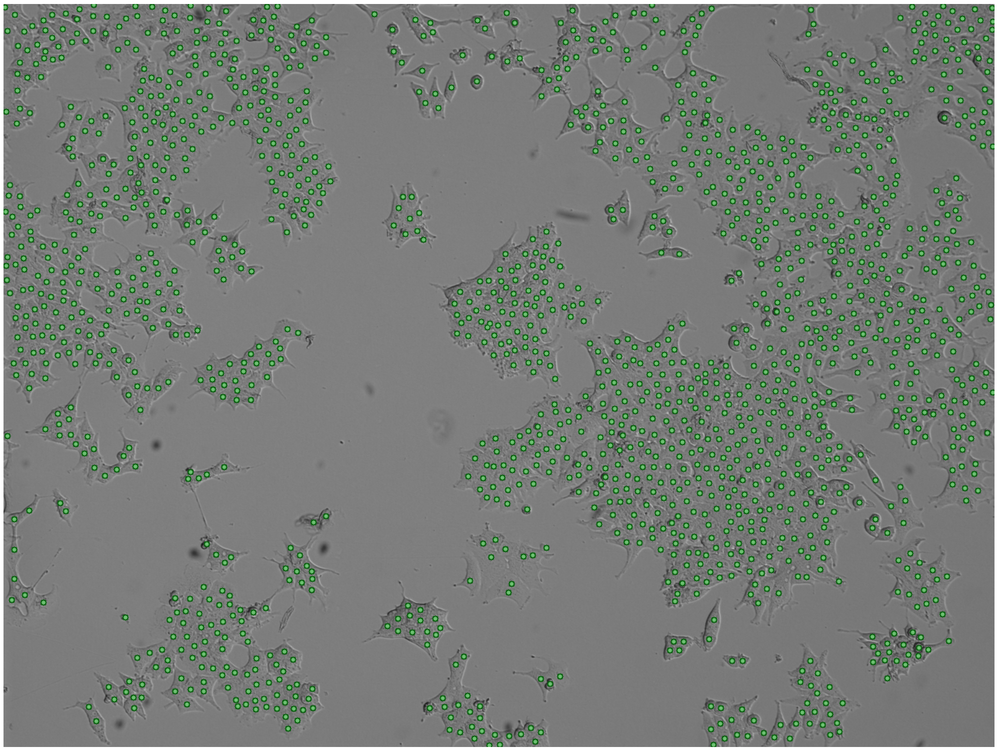

Supplementary Figure 9: 22Rv1 detections in crowded population (after domain adaptation).

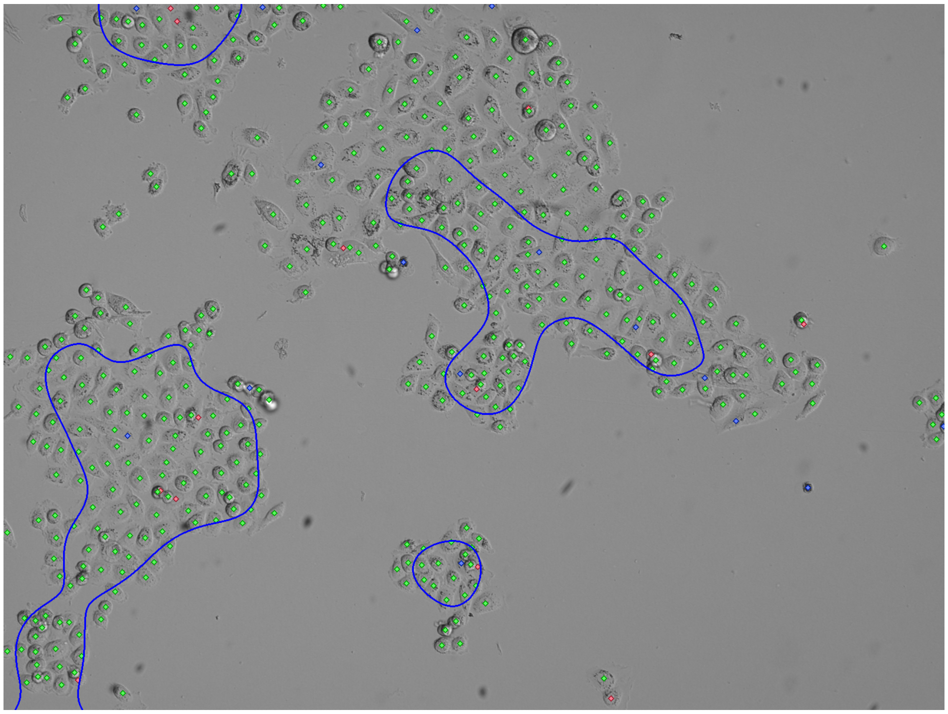

Supplementary Figure 10: Density area contours example with PC-3. Detections are marked as follows: green is for TP, red is for FN and blue is for FP. Same markings are used in following images.

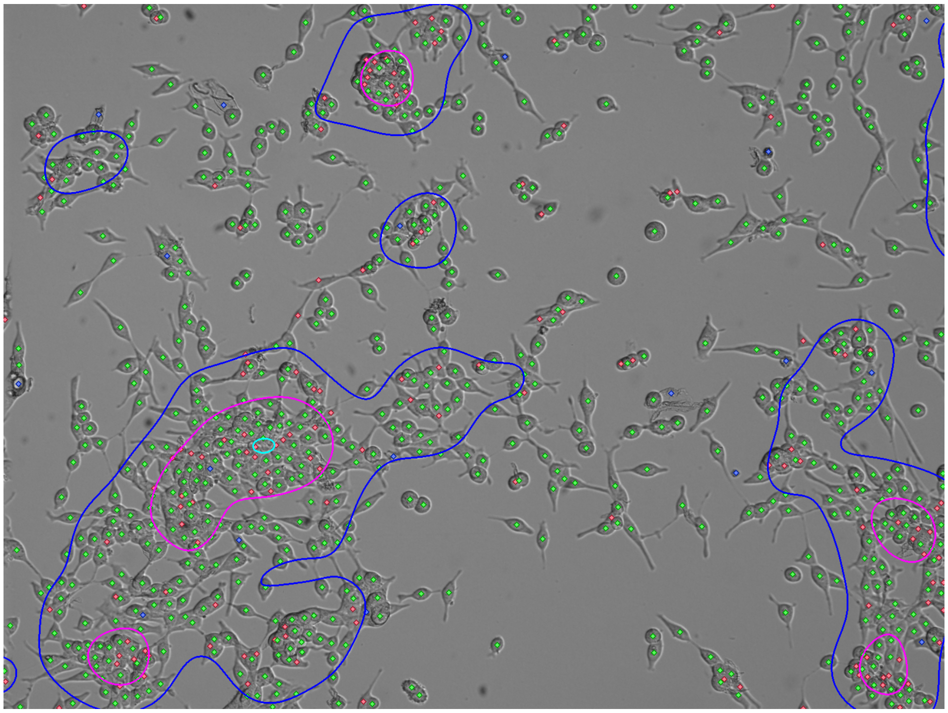

Supplementary Figure 11: Density area contours example with LNCaP.

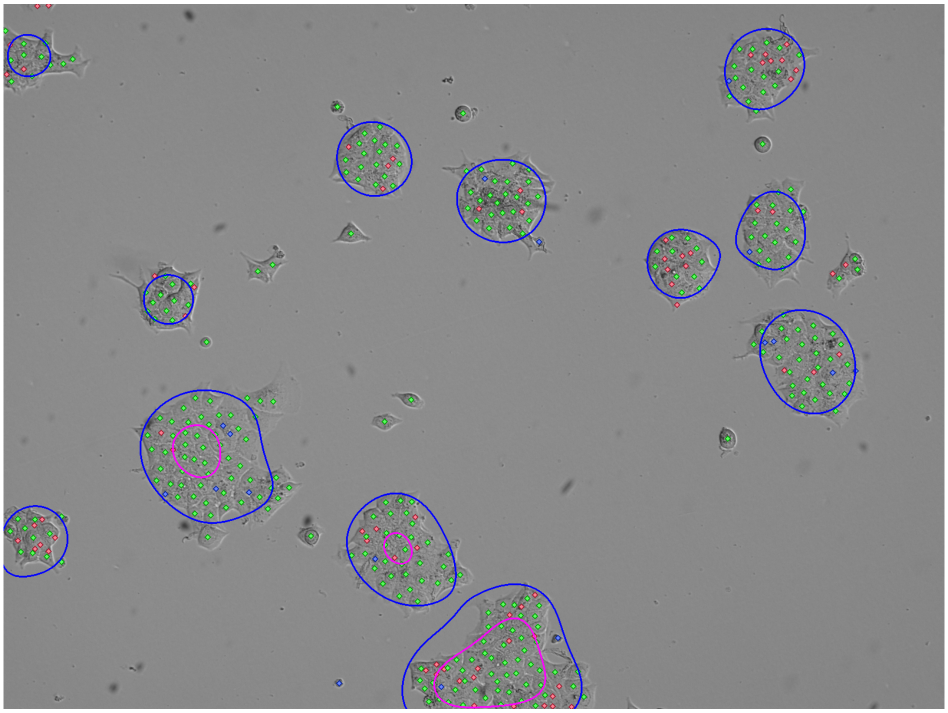

Supplementary Figure 12: Density area contours example with BT-474.

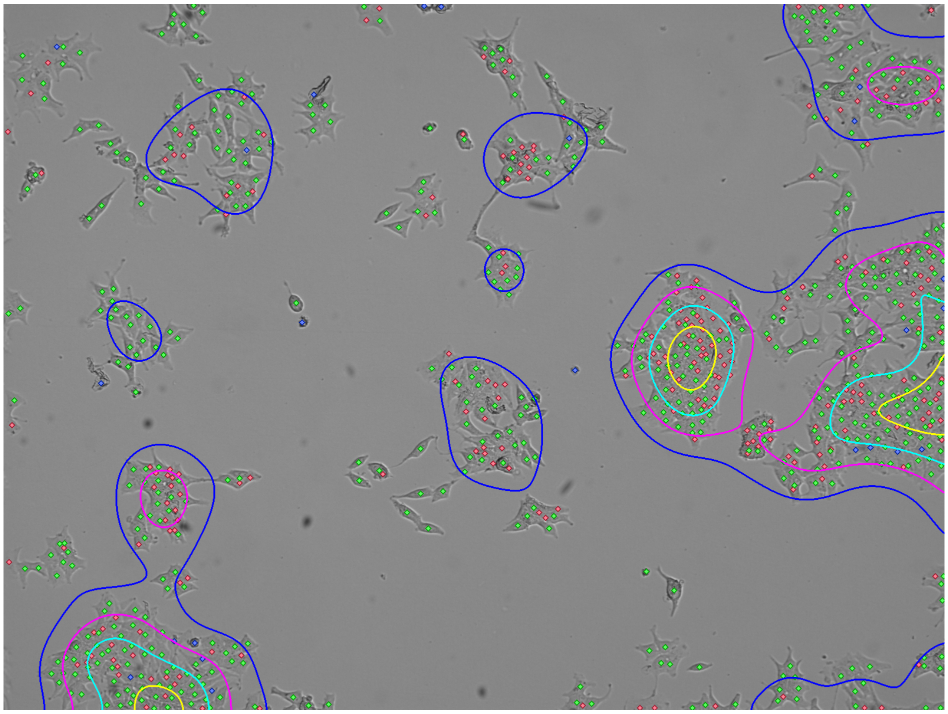

Supplementary Figure 13: Density area contours example with 22Rv1.
